# Implementing telephone triage in general practice: a process evaluation of a cluster randomised controlled trial

**DOI:** 10.1186/s12875-015-0263-4

**Published:** 2015-04-10

**Authors:** Jamie Murdoch, Anna Varley, Emily Fletcher, Nicky Britten, Linnie Price, Raff Calitri, Colin Green, Valerie Lattimer, Suzanne H Richards, David A Richards, Chris Salisbury, Rod S Taylor, John L Campbell

**Affiliations:** School of Health Sciences, Faculty of Medicine and Health Sciences, University of East Anglia, Norwich, UK; Primary Care Research Group, University of Exeter Medical School, Exeter, UK; Institute of Health Service Research, University of Exeter Medical School, Exeter, UK; Centre for Academic Primary Care, School of Social and Community Medicine, University of Bristol, Bristol, UK

**Keywords:** Telephone, Triage, General practice, Primary health care, Process evaluation, Qualitative interviews

## Abstract

**Background:**

Telephone triage represents one strategy to manage demand for face-to-face GP appointments in primary care. However, limited evidence exists of the challenges GP practices face in implementing telephone triage. We conducted a qualitative process evaluation alongside a UK-based cluster randomised trial (ESTEEM) which compared the impact of GP-led and nurse-led telephone triage with usual care on primary care workload, cost, patient experience, and safety for patients requesting a same-day GP consultation.

The aim of the process study was to provide insights into the observed effects of the ESTEEM trial from the perspectives of staff and patients, and to specify the circumstances under which triage is likely to be successfully implemented. Here we report perspectives of staff.

**Methods:**

The intervention comprised implementation of either GP-led or nurse-led telephone triage for a period of 2-3 months. A qualitative evaluation was conducted using staff interviews recruited from eight general practices (4 GP triage, 4 Nurse triage) in the UK, implementing triage as part of the ESTEEM trial. Qualitative interviews were undertaken with 44 staff members in GP triage and nurse triage practices (16 GPs, 8 nurses, 7 practice managers, 13 administrative staff).

**Results:**

Staff reported diverse experiences and perceptions regarding the implementation of telephone triage, its effects on workload, and on the benefits of triage. Such diversity were explained by the different ways triage was organised, the staffing models used to support triage, how the introduction of triage was communicated across practice staff, and by how staff roles were reconfigured as a result of implementing triage.

**Conclusion:**

The findings from the process evaluation offer insight into the range of ways GP practices participating in ESTEEM implemented telephone triage, and the circumstances under which telephone triage can be successfully implemented beyond the context of a clinical trial. Staff experiences and perceptions of telephone triage are shaped by the way practices communicate with staff, prepare for and sustain the changes required to implement triage effectively, as well as by existing practice culture, and staff and patient behaviour arising in response to the changes made.

**Trial registration:**

Current Controlled Trials ISRCTN20687662. Registered 28 May 2009.

## Background

Telephone triage represents one strategy which might be introduced in an attempt to manage demand for face-to-face GP appointments in primary care. There is an increasing body of evidence about the impact of telephone triage on GP workload [1], patient safety [1-5] and satisfaction [6-8], costs to healthcare [6], and patient-provider communication [9,10]. However, this evidence does not provide clear support or opposition for the implementation of telephone triage in primary care, and there is thus a key gap in understanding the circumstances under which telephone triage might be successful. This evidence is needed to assist GP practices to assess how their existing organisational structure and culture predisposes them to its successful implementation.

Telephone triage in primary care comprises numerous components, behaviours, targets and outcomes, all potentially impacting on providing individualised patient care in diverse practice environments. Evaluating such interventions is acknowledged to be challenging [11] but also necessary to identify and support best practice [12,13]. Process evaluation is an approach used to understand how complex health interventions such as triage are perceived, understood and delivered within the context of randomised controlled trials. The approach may utilise both quantitative and qualitative methods, and has the potential to provide important explanations for observed effects between different study arms [14]. The value of qualitative methods within process evaluations lies in identifying the reasons for participants’ attitudes, actions and approaches to change [15-20], and improving external validity beyond trial findings [21].

The research reported here was a process evaluation embedded within a multicentre three armed cluster randomised controlled trial (ESTEEM [1,6]) which compared effects on primary care workload, cost, patient experience of care, patient safety and health status of computer-supported nurse-led telephone triage, GP-led telephone triage, and usual care. The ESTEEM findings revealed that both nurse triage and GP triage led to an increased rate of primary care contacts over 28 days, compared with usual care, including the initial triage contact [1]. While the trial results did not support a clear recommendation encouraging a move to adopt GP triage or nurse triage for managing same-day appointments across the NHS, both forms of triage were found to be cost-equivalent with usual care, nurse-led triage led to an overall reduction in patient-GP contacts on the index day of contact (the day patients contacted their surgery to request a same-day appointment), and that therefore, triage may offer a useful approach to support flexible delivery of patient care.

The process evaluation for ESTEEM aimed to offer insights into the observed effects of introducing GP- or nurse-led telephone triage for patients seeking same-day appointments with a GP and to specify the circumstances under which triage is likely to be successfully implemented. To address these aims, the objectives were to describe how the telephone triage interventions were implemented in different practice settings, to describe the experience and acceptability of telephone triage for primary care staff and patients, and to elicit patient and staff views on what influences whether the telephone triage is seen to work or not work. Here, we report on the findings resulting from staff interviews conducted in practices implementing either GP-led or nurse-led triage (patient views are reported elsewhere [6]).

## Methods

### Participants and procedure

ESTEEM recruited 21,000 patients requesting same-day appointments in 42 General Practices across four different regions of England. Practices randomised to one of the two triage arms typically ran the intervention for a period of 2-3 months.

The GP and nurse triage interventions were complex interventions that involved staff training (clinical and technology based); a computer decision support software (‘Odyssey’ CDSS) to support the delivery of nurse triage; process and organisational change in practices regarding reception activity and appointment system management; process and organisational change in practices regarding reception activity and appointment system management; and accommodation of patient expectations. Some core elements of triage delivery were common to, and adopted by, all practices in both intervention arms. However, some organisational flexibility was permitted because of the complex nature of the intervention.

All patients contacting the practice initially spoke to a receptionist. Once the receptionist established that the patient (or a proxy asking on their behalf) was requesting a same-day, face-to-face appointment with a GP, the patient was asked to provide a contact telephone number and was advised that the clinician (GP or nurse, according to the practice’s allocation) would call them back within around 1–2 hours. This timescale was suggested as a guide for practices but was not considered mandatory.

The process evaluation was conducted during the implementation of the main trial, in a purposive sample of eight practices implementing a triage intervention, nurse-led or GP-led, across three regions (Devon, Bristol and Warwickshire), with a variety of list sizes and locations (inner-city, urban, suburban or rural; Table 1). A full outline of our approach to practice recruitment is provided elsewhere [1]. Staff were recruited to be interviewed about their perceptions and experience of triage within the ESTEEM Trial. Multi-centre research ethics approval (MREC) was obtained from South West 2 NHS Research Ethics Committee (REC) in October 2009 (reference 09/H0202/53). Written informed consent to be interviewed was obtained from all interviewees.

**Table 1:** **Overview of the process evaluation practices**

	**Triage type**	**List size of practice and location**	**Triage sessions (mode of delivery)**	Average (Stdev) number of triage calls completed by practice per day*	Overview of reported triage experience**
**Practice 1**	Nurse	9215 Suburban	Two nurses in morning only; one at a time	17.41 (6.98)	Very negative
**Practice 2**	Nurse	11267 Suburban	Two nurses each morning; in parallel	32.21 (5.91)	Quite positive
**Practice 3**	Nurse	5949 Rural	One nurse, morning & afternoon	6.03 (2.81)	Positive
**Practice 4**	Nurse	7981 Suburban	1-2 nurses morning & afternoon, switching to morning only	10.32 (3.81)	Positive
**Practice 5**	GP	5500 Rural	All GPs for 1.5 hrs in morning only; followed by duty triage GP	14.50 (4.68)	Mixed
**Practice 6**	GP	10622 Urban	One duty GP at a time on rota, also seeing triaged patients	8.88 (3.97)	Mixed
**Practice 7**	GP	11098 Suburban	Pre-surgery triage for all GPs, followed by duty doctor	34.08 (13.65)	Mainly positive
**Practice 8**	GP	5927 Urban	Duty doctor, on a rota	7.05 (3.90)	Positive

*Number of triage calls made by practice is not equivalent to number of same-day appointment requests received. A proportion of requests would have met the ESTEEM exclusion criteria^1^ and were therefore not included on the list of patients to call back.

**The classification of practices in this category was based on the summary of participant responses for each practice produced as part of the framework analysis.

#### Staff participants

All practice staff within sampled practices were invited to participate in the process evaluation; those who responded to the invitation and went on to participate were self-selected. A purposive sampling strategy was designed, selecting potential staff interviewees for occupational diversity to ensure that the views of GPs, nurses, practice managers and reception staff were represented (Table 2). Staff members of both sexes and of various ages were approached.

**Table 2:** **Practice staff participating in process evaluation**

	**GPs**	**Practice nurses or nurse practitioners**	**Managers**	**Administrative staff**	**Total**
**GP triage**	9	2	4	7	22
**Nurse triage**	7	6	3	6	22

### The interviews

Qualitative semi-structured interviews of 30-60 minutes’ duration were held with selected practice staff and conducted face-to-face within practice premises. Interviews explored preparation and training for ESTEEM; expectations, experiences and views held by staff of setting up and running triage; acceptability of triage to staff, problems, solutions and staff members’ hypothetical willingness to use triage following completion of the study. Interviews were conducted by LP and audio-recorded with the permission of interviewees.

### Analysis

The 44 interview audio-tapes were transcribed verbatim, checked and then anonymised. Transcripts were analysed thematically, drawing on grounded theory techniques of constant comparison [22] using the qualitative data analysis software package QSR NVivo 8. A deductive coding frame based on the process evaluation research questions was agreed by NB and LP. Within that coding frame, data were coded inductively to allow participants’ accounts to inform the analysis and to capture the subjectivities of a multiplicity of experiences in a range of settings. Inductive coding allowed prior themes within the coding frame to be extended or refuted. A framework approach [23] was adapted to enable both within-case (individual practices) and cross-case (between practices) analysis. Interviews were interrogated in terms of any areas of emerging agreement or disagreement about what worked, or did not work, and any observable conflicts and differences of opinion between and within staff groups. Coding and analysis was undertaken by one researcher (LP) and validated by a second researcher (NB), who reviewed a sample of the transcripts and tested these against the coding frame.

## Results

Several key themes emerged of factors which affected the experience of triage, and whether triage was found to be acceptable. These can be viewed in terms of how practices implemented triage, how staff resources were allocated to manage triage and the subsequent implications for roles and daily activities of practice staff, and how the introduction of the triage system was communicated to staff.

### Implementation of telephone triage intervention

Practices were advised by the study team to introduce triage within set blocks of time (i.e. whole morning or afternoon sessions) each day, and all did so. Most practices triaged in the mornings, although some had both morning and afternoon triage sessions – see Table 1.

#### Nurse-led triage

Two practices triaged only in the mornings, while the other two also included afternoon triage sessions. Nurses triaging simultaneously varied from one to three, and two practices provided extra nursing capacity for ESTEEM. Although the CDSS was regarded as a useful training tool by some nurses with no triage experience; more experienced nurses chose not to use it, or ignore its suggested actions, judging the CDSS to be unsuitable for use in daytime primary care.

I think the software isn’t necessarily the best for primary care. It’s very good for out of hours but primary care it’s not. There’s gaps in it. For instance if someone rings up to have a mole looked at, as one of the examples, there’s nothing on the Odyssey system that deals with moles. At all. Not at all. Once you know what it doesn’t accept, I haven’t used Odyssey at all on a few on them.

*Practice 1, Nurse Triage, Nurse*

Timing of triage was an issue in some nurse triage practices because doctors’ clinics coincided with triage times, meaning patients sometimes had insufficient time to travel to the surgery, especially those dependent on using public transport. This had the potential to leave appointment slots unfilled. The system was reported as problematic on Friday afternoons when, in general, practices tried to ensure patients were seen before the weekend.

#### GP-led triage

There were two main organisational structures for GP triage; a duty triage doctor system where GPs took turns to provide a dedicated triage service, and an integrated system where triage (usually of GPs’ own patients) was combined with normal surgery. Both systems presented issues of workload disparity between doctors. The duty triage system meant that GPs on triage duty on busy mornings, especially Mondays, struggled to get through the triage list. Disparities in the integrated system resulted from extra demand falling on certain GPs. For example, some female GPs faced extra demand from female patients with certain conditions; some GPs were ‘more popular’ than others, while other GPs had a ‘more challenging’ or ‘more demanding’ patient list.

The duty triage system had the additional challenge that the period when the duty doctor was triaging meant time lost to surgery, thus reducing the number of face-to-face appointments available. This placed an additional burden on the GPs not on triage duty, who then had to work extra sessions or take paperwork home. Demand was also uneven and unpredictable with attendant workload effects.

Because some days there’s about two pages, on a Monday or whatever, some days. You never know, do you, what days are going to be busy in general practice. And some days you think, “Oh look at that list, it’s just pages!”

*Practice 8: GP Triage, Practice Manager*

In practices operating a personal list system, the close doctor-patient relationship was considered to represent good care. Restricted access to the GP for his/her own patients was one of the consequences of triage that GPs felt would be detrimental to good relationships:

Yeah and we found the effect on our bookable appointments really embarrassing. Because we’re very committed to quality and looking after our patients and for people to be phoning up and saying you know, being told there’s no appointments for a month for your own doctor, you know, we didn’t tolerate that at all.

*Practice 7, GP triage, GP*

Two practices (Practices 5 and 7) combined a duty doctor system with pre-surgery triage in which non-duty doctors telephoned their own patients from the triage list before the duty doctor began triaging. Where an appointment was necessary, patients were allocated to an appointment with the triaging duty doctor, to their ‘own’ doctor, or to the first available appointment, depending on the practice.

### Allocation of staff resources and impact on roles of practice staff

A key feature of the success of triage and how staff perceived triage was how practices allocated staff to implement triage and conduct the trial. Practices delivering nurse triage generally identified that extra resources were required to support triage so as not to place an excessive burden on existing nurse workload.

Nurse (N): Yes. Right, I think for us, a big practice we definitely needed two nurses because we tried it with only one and it was just woah, suddenly by eleven you had so many calls to make it was, that was really stressful. So we quickly changed it to two nurses-

Interviewer (I): Right so you started off with one-

N: We couldn’t have coped with only one. We started with one and by the time we got to half past ten in the morning you’d be having a heart attack.

I: Right.

N: Because you suddenly looked and you had like 50 calls to make and it was going on till one o’clock, and then you start a proper clinic again at two.

I: So a similar size practice thinking of nurse triage, would you advise them to increase their nursing capacity really?

N: Well I think if you’re going to have a large practice and you’re going to have a nurse led triage you have to think about increasing your, definitely because you’re losing appointments aren’t you?

*Practice 2: Nurse Triage, Triaging Nurse*

In Practice 1, a large suburban practice, extra nurses were not provided and the perception of triage by the triaging nurse and reception staff was invariably negative. However, regardless of whether extra nursing staff was put in place, it was frequently reported that triage impacted upon the availability of routine nurse appointments:

I: No. What’s it (triage) stopping you from doing then?

N: Well doing my asthma reviews, my COPD reviews. L and I used to see a few same day appointments which would come through the door, you know and you know all these run of the mill practice nursing stuff. Therefore a lot of that’s been put on hold.

I: Yes, yeah so on hold for a while is fine, but permanently?

N: No, we’d have to have an extra nurse in I think, which would defeat the object because I think the whole point is to try and save money and it wouldn’t do. No, no.

*Practice 1: Nurse Triage, Triaging Nurse*

Despite this negative perception that nurse triage was not cost-effective, GPs in Practice 1 appeared to have a positive perception of triage, which they linked to a reduction in GP workload, supporting the main trial findings [1].

I: So how about your other colleagues within the practice here, your other GP colleagues, how have they responded to the triage?

GP: Pretty well actually and I think it’s been useful to, well certainly on a Monday when I work I’ve found it a real positive benefit having the triage because I think there has been, they have managed to take some of the work load off us which has been good. And I think partly because we are down on doctors because these doctors that have left, it’s meant that we’ve probably just about coped with one less doctor on the days. Probably about a quarter of our resource that we normally have on that day is not there. So it has had a positive effect.

*Practice 1: Nurse Triage, GP*

As these extracts demonstrate, clinicians frequently viewed the benefit of triage in terms of the impact on the workload of their own professional group. In nurse triage practices, triage also had consequences for how nurses organised their working shifts to ensure patients could be booked into available appointments with GPs:

Yeah. I was at one point coming in … I do Monday and a Tuesday afternoon half-past-three to half-past-six and there was an issue whereby I was coming in early. I was coming in at about three o’clock off my own back. Because one of the doctors here finished at four. So if I didn’t come until half-past-three and then start triaging I’d miss all her ESTEEM. Because she would have like three or four ESTEEM patient slots at the end. I can’t phone a patient, “Right, your appointment’s actually now. Can you get here now?” You can’t do that. So then our … then all the ESTEEM patients were coming to the doctor … left the doctor who was on until half-past-five and then either you ran out of ESTEEM slots or they were ridiculously busy. So the doctor who was on two until four or whatever it was kind of had like four ESTEEM slots free at the end of the surgery because the nurse wasn’t here in time to catch them.

*Practice 3: Nurse Triage, Nurse*

In contrast to the consequences of under-resourcing nurse triage, the introduction of GP triage had a different impact on the nursing role in one practice where the nurse practitioner had previously triaged patients face-to-face:

I haven’t liked it at all I have to say, and as it goes on and on and on, I’m liking it less and less and less. I joked at the beginning when we went onto this trial, you know, I laughed and said oh well, I won’t be doing anything. I’m going to take 12 weeks off and everybody said “Oh, you’ll be busy, you’ll be busy.” I haven’t been busy and it drives me nuts… They talk about having a salaried GP when I go because they think it will be better in that they can do everything that a doctor can do. But what they don’t seem to have taken on board and they, I’m not sure who it is, they don’t realise that actually, I pick up quite a lot of work that a GP won’t do.

*Practice 6 – GP Triage, Nurse Practitioner*

From the perspective of the nurse practitioner reported in the quote above, introducing GP triage removed a central activity within her everyday work. Similarly, for other nurses, delivering nurse triage was viewed as a move away from employing their clinical skills to conduct face-to-face chronic illness reviews, to a role as remote gatekeeper to GP care, a view sometimes shared with administrative staff:

They [nurses] hate it. They absolutely hate it. They say, you know, “we trained to be a practice nurse not a telephone operator.” And they’ve even said if they take it up then they’ll be looking for other jobs because this is not what they trained to do.

*Practice 1: Nurse Triage, Lead Administrator*

However, there was also evidence that nurse triage, using CDSS could have a positive impact on nurses’ skills:

I’ve quite enjoyed it, it’s been quite challenging, you know, and it’s made me think a bit more and also it’s actually made me in some ways confirm that what I’m doing is correct and what I think I’ve thought I was doing was correct. And obviously it’s given me some further advice on things and maybe think a bit more about things, which I may have forgotten or are new to me. So it’s been a learning curve and it’s again quite a sense of an achievement when you actually finish the morning, phew you know, *(laughter)* I’ve done it and I’ve got through and there haven’t been any dramas, calamities, you know.

*Practice 4: Nurse Triage, Nurse 1*

Completing a triage session without any ‘calamities’ highlights how, for some nurses, telephone triage represented a challenge to delivering safe care:

It’s very hard to assess people over the phone sometimes, trying to gauge and I think it’s good to keep going into it fresh. And it has been good but sometimes when you’ve busy day on top of busy day, and you are, just with anything I suppose you feel the pressure a little bit because you worry about those people who you didn’t bring in or you worry that the GP might be annoyed with you about people that you did bring in.

*Practice 4: Nurse Triage, Nurse 2*

These examples emphasise a reconfiguration of skills, activity and function within working practice, which was not limited to nurses, but could also be seen in how administrative staff perceived and reported their own role:

It [GP triage] is brilliant, it’s absolutely brilliant. Because I think the worst part of my job and any of the receptionists that you ask, has always been that there’s never enough appointments to go around for the people that want them. So you’re already… So we then have to, would have to triage them and put them on the end of the doctor’s surgery if they’re urgent which puts the onus on us which… So now that it’s swapped around, it’s brilliant.

*Practice 5: GP Triage, Receptionist*

Implementing triage therefore requires careful consideration not only of resources needed to be in place, how the practice will be reorganised, and the skills needed to deliver triage effectively, but also the impact triage will have on how different staff perceive their role. Communicating plans to implement triage and involving all staff in the decision-making process is therefore vital to shaping these perceptions.

### Communication across practice staff

In Practice 1, there was generally a negative perception of triage which could be seen to be linked to how nurses felt their roles had been reconfigured as a result of participating in the trial.

And an awful lot of my time and L’s time is spent speaking to people on the phone, which is not actually saving anything but taking up time when we could actually be seeing some of these patients.

*Practice 1, Nurse Triage, Nurse*

These views were also reflected in how administrative and nursing staff perceived the decision to participate in ESTEEM was made and communicated to them.

They didn’t ask me who would do the appointments and how it would be, and it was just this is what we’re doing. And they don’t listen to the people that know. It is a shame really.

*Practice 1, Nurse Triage, Lead Administrator*

This perception was reinforced when a GP in the same practice was asked about the decision to participate in the study:

I: And you’ve been involved in the practice joining the trial?

GP: Yes, from a policy perspective I’m the executive partner and so I have a hand in the decision that we wish to be involved with it. And I have a personal interest in the different ways of accommodating the demands that we are placed under and managing the resources that we have to deploy them to best effect.

I: Your nursing colleagues, have you had any feedback from them about how they found the process?

GP: No, we are short staffed at the moment so I don’t talk to them.

*Practice 1, Nurse Triage, GP*

In contrast, the Practice Manager in Practice 5 reported a number of strategies to help ensure staff adjusted to the introduction of triage, including holding regular meetings with staff, listening in to calls between patients and receptionists, acknowledging challenges faced by staff, and expressing gratitude for tackling these challenges:

It was mainly about face-to-face meetings and picking up when you could see people were flagging. And also sitting in downstairs…when I sit in the office sometimes between when I first get in until my…I might stay down there for just half an hour, three quarters of an hour and listening to the calls, and you can hear that there’s sort of really challenging conversations that some of them are having to have with people who haven’t heard about the system, don’t understand it, don’t want it and [the] incredibly patient response that the receptionists are giving…I’m just constantly thanking them really and saying how well they’re doing and how well they’ve handled certain calls that I can hear have been really difficult.

*Practice 5, Practice Manager*

How staff viewed and responded to triage could therefore have been influenced by how effectively and inclusively its aims and intended effect on patient care were communicated by those making the decision to implement it, as well as the support provided to manage change.

## Discussion

The quantitative findings from the ESTEEM trial [1] in which the process evaluation was embedded, revealed that both nurse triage and GP triage led to an increased rate of primary care contacts over 28 days, compared with usual care. The findings of this process evaluation offer insight into the reasons why telephone triage was responded to positively by staff in some practices and negatively within others, as well as indicating how telephone triage may offer a useful approach to support flexible delivery of patient care.

The considerable variation in the degree to which triage was judged acceptable, both between and within practices echoes a qualitative study of the Advanced Access model of telephone triage [24], which also found wide variation in interpretation and implementation of the model, and noted that informal organisational behaviour resulted in its adaptation to practice contexts, norms and values. Staff resources need to be appropriately allocated in order to ensure staff are not overburdened, and appointment sessions need to be organised in line with triage sessions to ensure that available appointments are not wasted or patients not inappropriately triaged into emergency slots.

The ESTEEM trial also found that on the index day of contact, nurse triage led to a 28% reduction in patient-GP contacts, a 31% reduction in GP face-to-face contacts, and a related reduction of 1.4 minutes in overall GP contact time [6]. This was a redistribution of workload from GPs to nurses which is reflected in the findings reported here, in particular helping to explain nurses’ negative perceptions of triage when additional resources were not put in place by practices. However, in contrast to some staff perceptions, nurse triage was found to be cost equivalent with both GP triage and usual care. Implementing nurse triage in a way which is acceptable to practice staff therefore requires careful consideration of the resources required to support nurse triage but also clear communication with practice staff regarding the consequential impact on practice workload and cost.

The decision to implement triage needs to be effectively communicated within the practice, with formal and informal opportunities for airing and sharing problems and successes. Where the criteria of good communication within the practice, supportive staff relations and full consultation with staff regarding trial participation were present, particularly if there was a culture of accepting change, triage was typically reported as acceptable by staff. However, for some staff, telephone triage challenged beliefs about what constitutes good patient care, in particular the axiom that seeing patients is an essential component of good, safe care. This was particularly evident for nurses who viewed telephone triage as limiting their use of clinical skills that were being deployed in face-to-face consultations.

Introducing telephone triage is not just a matter of ensuring the appropriate staff resources are in place. How staff perceived telephone triage was related to how they perceived their own role, how they perceived the value of their skills prior to introducing triage, and also how they perceived the skills required to conduct triage. Telephone triage reconfigures the function, skills and identities of staff according to how the activities of their everyday working practice shift as a result of its introduction.

Richards & Borglin [25] argue that the complexity of nursing is such that it can be seen as the ‘quintessential complex intervention’ – comprising a number of component parts which, when applied to a target population, has the potential to produce a range of possible outcomes. Our findings reflect this argument, nurses embark on telephone triage from a very different starting point from GPs, and embedding nurse triage into practice is likely to take longer than perhaps a GP triage system might, requiring additional preparation, support and resourcing. Implementing nurse triage in the absence of this preparation is unlikely to be as successful as a carefully managed change. Nurse triage is less likely to be successful if practices do not support nurses by ensuring there is sufficient nurse capacity to undertake this new role as well as performing their usual roles such as health promotion, chronic disease management, and vaccination programmes.

### Strengths and limitations of the study

The process evaluation has provided rich data on the experience of different models of triage, and how these models were adapted to local circumstances. Our findings demonstrated diversity in how the two models of triage investigated in the ESTEEM trial were implemented and perceived by staff. Such diversity has raised important issues that any practice considering implementing triage might want to reflect upon before proceeding. It has also raised important implications for the conduct of RCTs in complex settings, demonstrating that a trial protocol is contextually contingent and subject to interpretation and adaptation rather than being the fixed and immutable fulcrum on which the science is balanced. However, Hawe et al [26] argues that such variation is inevitable and permissible in community trials, as long as investigators are able to identify the active ingredients of complex interventions. Our findings reinforce this point and provide yet more support for the importance of including qualitative investigation of complex interventions alongside trials to maximise learning.

As is the case with all trials with an embedded process evaluation, it was not triage alone that was being evaluated, but triage introduced and delivered under trial conditions. This represents a consideration for practices considering using our data to inform the introduction of triage. The demands of the trial introduced confounding factors, additional pressures and strictures to be negotiated at the same time as introducing a major organisational change. Findings may also have been different if data had been collected at a different stage in the process, perhaps when staff had had time to become more accustomed to telephone triage and felt more competent and confident. Indeed, some participants contrasted what they or others felt ‘at first’ with how they felt at the time of the interview. Finally, we have not reported patient views in this article (reported elsewhere [6]), and it is important to acknowledge that staff views of triage would have been influenced by patients’ reactions to the introduction of triage. However, the findings reported here are of the challenges of implementing telephone triage from within the context of a trial, and from the perspective of NHS staff. Patients were not specifically advised on the context of the RCT and their views need to be set in a different context to the views of NHS staff.

## Conclusions

Introducing telephone triage in primary care requires careful consideration of the organisational structure and culture, staff skills and experience, perceived need and anticipated outcomes, staffing issues, and the geographical location and primary care setting in which triage might be introduced. There needs to be clear communication with all practice staff regarding the decision to introduce triage and the subsequent impact on practice workload and cost. Staff resources need to be appropriately allocated in order to ensure staff are not overburdened, and appointment sessions need to be organised in line with triage sessions to ensure appointments are not wasted and that patients are not being inappropriately triaged into emergency slots.

Primary care practice staff also have specific perceptions of their role and how their skills are best deployed. The introduction of telephone triage, with additional use of computer decision support software in nurse triage, will impact on this perceived function within everyday general practice. These considerations will be critical in how the decision to implement triage is reached, how staff are engaged in this process, how staff are supported through its introduction, and ultimately, in how successful telephone triage is upon its implementation.

## Abbreviations

CDSS: Computer decision support software
ESTEEM: UK-based cluster randomised trial which compared the impact of GP-led and nurse-led telephone triage with usual care on primary care workload, cost, patient experience, and patient safety for patients requesting a same-day GP consultation
